# Poly[[tri-μ_3_-hydroxido-tris­(μ_4_-pyridine-2,5-dicarboxyl­ato)trineodymium(III)] monohydrate]

**DOI:** 10.1107/S160053681201286X

**Published:** 2012-04-04

**Authors:** Qing Zhang, Xing Wang, Shen-Tang Wang, Chun-Bo Liu, Guang-Bo Che

**Affiliations:** a School of Chemistry and Chemical Engineering, Jiangsu University, Zhenjiang 212013, People’s Republic of China

## Abstract

In the title compound, {[Nd_3_(C_7_H_3_NO_4_)_3_(OH)_3_]·H_2_O}_*n*_, the Nd^III^ atom is eight-coordinated by the three O atoms of three asymmetrically μ_3_-bridging hydroxide groups, by four carboxyl­ate O atoms of four different pyridine-2,5-dicarboxyl­ate (2,5-pydc) ligands, and by the N atom of a 2,5-pydc ligand. Six Nd atoms are connected by six hydroxide groups, forming an [Nd_6_(μ_3_-OH)_6_] cluster unit of symmetry -3 and a slightly compressed octa­hedral geometry. Adjacent [Nd_6_(μ_3_-OH)_6_] clusters are connected by the 2,5-pydc ligands, *via* O and N atoms, forming chains along the *c* axis. The remaining O atoms of the 2,5-pydc ligands link these chains into a three-dimensional framework. A disordered water molecule, located on a threefold rotation axis at the opposite side of the [Nd_6_(μ_3_-OH)_6_] cluster and exposed to each of the three Nd atoms, completes the structure.

## Related literature

For the importance of the 2,5-pyridine dicarboxylate ligand, see: Qin *et al.* (2005 ▶); Song *et al.* (2005 ▶); Huang, Jiang *et al.* (2008 ▶); Huang *et al.* (2007 ▶). For related coordination polymers involving 2,5-pyridine dicarboxylate ligands, see: Aghabozorg *et al.* (2008 ▶); Xu *et al.* (2008 ▶); Colak *et al.* (2010 ▶). For the use of compounds with *M*—O—*M* frameworks, see: Huang *et al.* (2007 ▶); Price *et al.* (2001 ▶); Huang, Song *et al.* (2008 ▶); Zhang *et al.* (2009 ▶). 
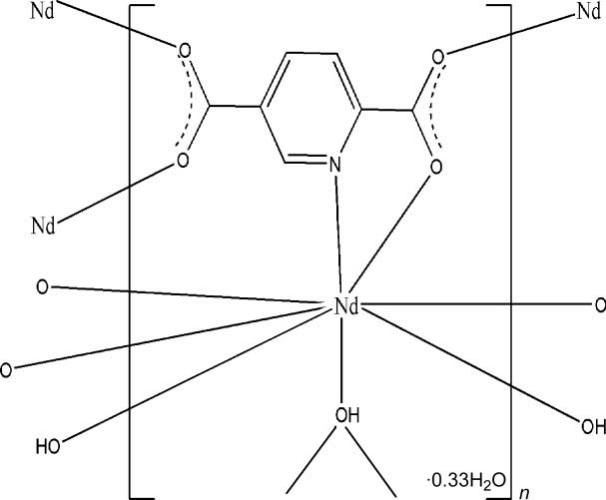


## Experimental

### 

#### Crystal data

[Nd_3_(C_7_H_3_NO_4_)_3_(OH)_3_]·H_2_O
*M* *_r_* = 997.07
Hexagonal, 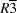

*a* = 23.081 (3) Å
*c* = 8.9690 (18) Å
*V* = 4138.0 (12) Å^3^
*Z* = 6
Mo *K*α radiation
μ = 5.65 mm^−1^
*T* = 297 K
0.16 × 0.15 × 0.11 mm

#### Data collection

Bruker SMART CCD area-detector diffractometer
Absorption correction: multi-scan (*SADABS*; Bruker, 2002 ▶) *T*_min_ = 0.421, *T*_max_ = 0.538
3454 measured reflections
1679 independent reflections
1558 reflections with *I* > 2σ(*I*)
*R*_int_ = 0.018

#### Refinement

*R*[*F* ^2^ > 2σ(*F* ^2^)] = 0.019
*wR*(*F* ^2^) = 0.043
*S* = 1.10
1679 reflections
133 parameters
H atoms treated by a mixture of independent and constrained refinement
Δρ_max_ = 0.77 e Å^−3^
Δρ_min_ = −0.65 e Å^−3^

### 

Data collection: *SMART* (Bruker, 2002 ▶); cell refinement: *SAINT* (Bruker, 2002 ▶); data reduction: *SAINT*; program(s) used to solve structure: *SHELXS97* (Sheldrick, 2008 ▶); program(s) used to refine structure: *SHELXL97* (Sheldrick, 2008 ▶); molecular graphics: *SHELXTL* (Sheldrick, 2008 ▶); software used to prepare material for publication: *SHELXTL*.

## Supplementary Material

Crystal structure: contains datablock(s) global, I. DOI: 10.1107/S160053681201286X/qk2031sup1.cif

Structure factors: contains datablock(s) I. DOI: 10.1107/S160053681201286X/qk2031Isup2.hkl

Additional supplementary materials:  crystallographic information; 3D view; checkCIF report

## Figures and Tables

**Table 1:** Selected bond lengths (Å)

Nd1—O2^i^	2.395 (2)
Nd1—O3^ii^	2.426 (2)
Nd1—O1^iii^	2.452 (2)
Nd1—O4	2.480 (2)
Nd1—O5	2.482 (2)
Nd1—O5^iv^	2.485 (2)
Nd1—O5^v^	2.501 (2)
Nd1—N1	2.747 (3)

Symmetry codes: (i) 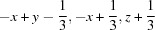; (ii) 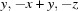; (iii) 
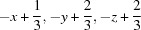
; (iv) 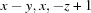; (v) 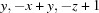.

**Fig. 1.**
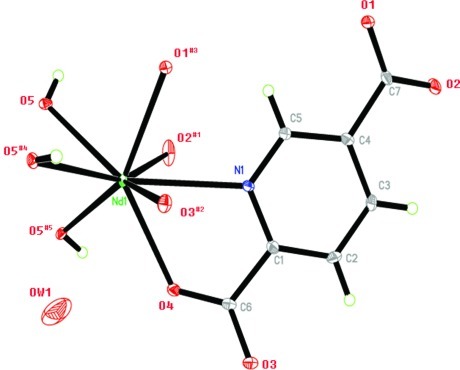
The asymmetric unit of tittle compound with the atom numbering scheme. Displacement ellipsoids are drawn at the 30% probability level (arbitrary spheres for the H atoms). (Symmetry codes: #1 -x+y-1/3,-x+1/3,z+1/3; #2 y,-x+y,-z; #3 -x+1/3,-y+2/3,-z+2/3; #4 x-y,x,-z+1; #5 y,-x+y,-z+1)

**Fig. 2.**
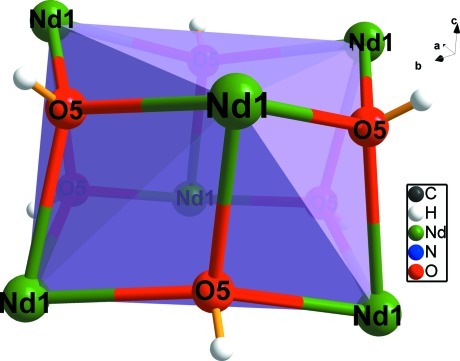
View of the hexanuclear [Nd6(µ3-OH)6] cluster unit in the title compound.

**Fig. 3.**
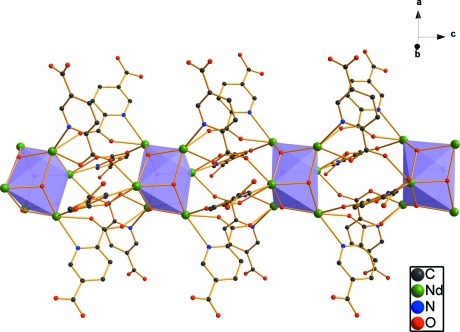
1-D chain structure of the [Nd6(µ3-OH)6] clusters linked by 2,5-pydc ligands along the c-axis. H atoms have been omitted.

**Fig. 4.**
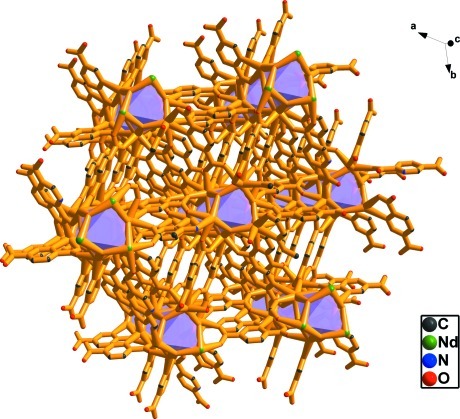
View of the three-dimensional structure of title compound linked by 2,5-pydc ligands. H atoms and O1w have been omitted.

## supplementary crystallographic information

### Comment

In recent years, much attention has been paid to the research on the coordination chemistry of 2,5-pyridinedicarboxylic acid (2,5-pydc), including complexes of lanthanide (Qin *et al.* 2005; Song *et al.* 2005; Huang *et al.* 2007; Huang, Jiang *et al.* 2008). The 2,5-pydc ligand acts as a good O donor as well as a N donor, owing to the two carboxylate groups and the pyridine ring, which may help to increase the dimensionality of the assembled covalent network (Aghabozorg *et al.* 2008; Xu *et al.* 2008; Colak *et al.* 2010). In addition, the construction of multidimensional M–O–M frameworks has been shown to produce materials with effective cooperation and has also lead to improvements in thermal stabilities (Huang *et al.* 2007; Price *et al.* 2001; Huang, Song *et al.* 2008; Zhang *et al.* 2009)

The title compound crystallizes in a trigonal lattice of space group symmetry R3. The neodymium atom is trivalent and is eight-coordinated by three oxygen atoms (O5, O5^iv^ and O5^v^) of three µ_3_-bridging hydroxyls, four carboxylate oxygen atoms (O1^iii^, O2^i^, O3^ii^ and O4) of four different 2,5-pydc ligands, and a nitrogen atom (N1) of a 2,5-pydc ligand as shown in Fig. 1. Each six Nd atoms are connected by six hydroxide groups to form a cluster unit [Nd_6_(µ_3_-OH)_6_] of symmetry 3 and with the shape of an octahedron slightly compressed along the threefold crystallographic axis (Fig. 2). The Nd-O bond lengths in this cluster vary from 2.482 (3) to 2.502 (3) Å, and the internal Nd···Nd distances are 4.018 (inclinded to the threefold axis) and 4.530 Å (perpendicular to the threefold axis). Such cluster units are linked by 2,5-pydc ligands *via* their O3, O4, and N1 atoms to form an extended single-chain structure as shown in Fig. 3. Neighbouring single chains are then connected by the O1 and O2 atoms of the 2,5-pydc ligands to form a three-dimensional network (Fig. 4). Each 2,5-pydc ligand acts as a µ_4_-bridge to link four Nd atoms, in which the nitrogen N1 and the oxygen O4 of the 2-carboxylate group chelate one Nd, while its other oxygen O3 ligates another Nd atom in monodentate mode. The 5-carboxylate group ligates two Nd atoms in dimonodentate fashion. The crystal structure is completed by a water molecule O1w, which is located on a threefold axis and has a pyramidal environment by three Nd at a distance of 2.984 (3) Å, which is about 0.5 Å longer than that of the coordination partners of Nd. The relatively large anisotropic displacement parameters of O1w indicate, that this molecule is disordered and that it probably deviates somewhat from the average position on the threefold axis.

### Experimental

All reagents were commercially available and used without any further purification. A mixture of 2,5-pyridine dicarboxylic acid (0.0167 g, 0.1 mmol), Nd(NO_3_)_3_.6H_2_O (0.0661 g, 0.2 mmol), 13 drops of 1 mol/L NaOH and distilled water (10 mL) was placed in a 25 mL Teflon-lined stainless steel autoclave, and heated at 453 K for 3 days. Cooling slowly to room temperature, the pink prism crystals of title complex were obtained.

### Refinement

All H atoms on C atoms were positioned geometrically (C—H = 0.93Å) and refined as riding, with *U*_iso_(H)= 1.2*U*_eq_(C). The H atom of the bridging hydroxy ligand O5 was located in a difference Fourier map and refined independently with *U*_iso_(H) = 1.5*U*_eq_(O). The hydrogen atoms of the water molecule O1w, which is located on a threefold axis, could not be located. According to an extra refinement, O1w is fully occupied but may deviate slightly from the threefold axis, as indicated by the relatively large displacement parameters of this atom.

### Crystal data

**Table:**

[Nd_3_(C_7_H_3_NO_4_)_3_(OH)_3_]·H_2_O	*D*_x_ = 2.401 Mg m^−^^3^
*M**_r_* = 997.07	Mo *K*α radiation, λ = 0.71073 Å
Hexagonal, *R*3	Cell parameters from 3338 reflections
Hall symbol: -R 3	θ = 3.1–29.0°
*a* = 23.081 (3) Å	µ = 5.65 mm^−^^1^
*c* = 8.9690 (18) Å	*T* = 297 K
*V* = 4138.0 (12) Å^3^	Prism, pink
*Z* = 6	0.16 × 0.15 × 0.11 mm
*F*(000) = 2814	

### Data collection

**Table:**

Bruker SMART CCD area-detector diffractometer	1679 independent reflections
Radiation source: fine-focus sealed tube	1558 reflections with *I* > 2σ(*I*)
Graphite monochromator	*R*_int_ = 0.018
ω scans	θ_max_ = 25.3°, θ_min_ = 3.1°
Absorption correction: multi-scan (*SADABS*; Bruker, 2002)	*h* = −17→26
*T*_min_ = 0.421, *T*_max_ = 0.538	*k* = −27→23
3454 measured reflections	*l* = −10→7

### Refinement

**Table:**

Refinement on *F*^2^	Primary atom site location: structure-invariant direct methods
Least-squares matrix: full	Secondary atom site location: difference Fourier map
*R*[*F*^2^ > 2σ(*F*^2^)] = 0.019	Hydrogen site location: inferred from neighbouring sites
*wR*(*F*^2^) = 0.043	H atoms treated by a mixture of independent and constrained refinement
*S* = 1.10	*w* = 1/[σ^2^(*F*_o_^2^) + (0.021*P*)^2^] where *P* = (*F*_o_^2^ + 2*F*_c_^2^)/3
1679 reflections	(Δ/σ)_max_ = 0.001
133 parameters	Δρ_max_ = 0.77 e Å^−^^3^
0 restraints	Δρ_min_ = −0.65 e Å^−^^3^

### Special details

**Table:**

Geometry. All esds (except the esd in the dihedral angle between two l.s. planes) are estimated using the full covariance matrix. The cell esds are taken into account individually in the estimation of esds in distances, angles and torsion angles; correlations between esds in cell parameters are only used when they are defined by crystal symmetry. An approximate (isotropic) treatment of cell esds is used for estimating esds involving l.s. planes.
Refinement. Refinement of F^2^ against ALL reflections. The weighted R-factor wR and goodness of fit S are based on F^2^, conventional R-factors R are based on F, with F set to zero for negative F^2^. The threshold expression of F^2^ > 2sigma(F^2^) is used only for calculating R-factors(gt) etc. and is not relevant to the choice of reflections for refinement. R-factors based on F^2^ are statistically about twice as large as those based on F, and R- factors based on ALL data will be even larger.

### Fractional atomic coordinates and isotropic or equivalent isotropic displacement parameters (Å^2^)

**Table:**

	*x*	*y*	*z*	*U*_iso_*/*U*_eq_	
Nd1	0.041666 (9)	0.128240 (9)	0.329953 (18)	0.00891 (7)	
C1	0.03699 (16)	0.21892 (16)	0.0150 (4)	0.0131 (7)	
C2	0.0445 (2)	0.26676 (18)	−0.0868 (4)	0.0253 (9)	
H2	0.0244	0.2544	−0.1801	0.030*	
C3	0.0822 (2)	0.33329 (18)	−0.0484 (4)	0.0290 (10)	
H3	0.0873	0.3664	−0.1150	0.035*	
C4	0.11239 (17)	0.35025 (16)	0.0903 (4)	0.0157 (7)	
C5	0.10166 (17)	0.29858 (16)	0.1863 (4)	0.0137 (7)	
H5	0.1211	0.3095	0.2804	0.016*	
C6	−0.00545 (16)	0.14569 (16)	−0.0166 (4)	0.0121 (7)	
C7	0.15650 (17)	0.42256 (17)	0.1364 (4)	0.0150 (8)	
N1	0.06494 (13)	0.23415 (13)	0.1504 (3)	0.0113 (6)	
O1	0.19772 (12)	0.43481 (11)	0.2386 (3)	0.0186 (6)	
O2	0.14805 (13)	0.46471 (12)	0.0684 (3)	0.0261 (6)	
O3	−0.02355 (11)	0.12927 (11)	−0.1493 (2)	0.0147 (5)	
O4	−0.02057 (12)	0.10561 (11)	0.0918 (3)	0.0167 (5)	
O1w	0.0000	0.0000	0.1697 (6)	0.0634 (18)	
O5	0.03467 (11)	0.10550 (12)	0.6017 (3)	0.0118 (5)	
H1	0.0397 (19)	0.1334 (18)	0.650 (5)	0.018*	

### Atomic displacement parameters (Å^2^)

**Table:**

	*U*^11^	*U*^22^	*U*^33^	*U*^12^	*U*^13^	*U*^23^
Nd1	0.00801 (11)	0.01015 (11)	0.00950 (11)	0.00524 (8)	0.00187 (7)	0.00291 (7)
C1	0.0133 (17)	0.0111 (17)	0.0147 (17)	0.0060 (15)	−0.0010 (14)	−0.0013 (14)
C2	0.037 (2)	0.019 (2)	0.0163 (18)	0.0109 (18)	−0.0151 (17)	−0.0036 (17)
C3	0.043 (3)	0.0125 (19)	0.024 (2)	0.0085 (19)	−0.0149 (19)	0.0048 (16)
C4	0.0184 (19)	0.0104 (17)	0.0162 (18)	0.0057 (15)	−0.0035 (15)	−0.0027 (15)
C5	0.0146 (17)	0.0117 (17)	0.0134 (17)	0.0056 (15)	−0.0029 (14)	−0.0012 (14)
C6	0.0075 (16)	0.0135 (17)	0.0136 (17)	0.0039 (14)	0.0005 (14)	−0.0043 (14)
C7	0.0141 (17)	0.0104 (17)	0.0154 (18)	0.0023 (15)	0.0022 (15)	0.0008 (15)
N1	0.0113 (14)	0.0098 (14)	0.0115 (14)	0.0044 (12)	−0.0018 (12)	−0.0018 (11)
O1	0.0217 (14)	0.0120 (12)	0.0182 (13)	0.0055 (11)	−0.0091 (11)	−0.0003 (10)
O2	0.0294 (15)	0.0104 (13)	0.0324 (15)	0.0054 (12)	−0.0165 (13)	0.0040 (12)
O3	0.0144 (12)	0.0151 (13)	0.0132 (12)	0.0063 (10)	−0.0013 (10)	−0.0043 (10)
O4	0.0180 (13)	0.0114 (12)	0.0120 (12)	0.0008 (11)	0.0006 (10)	−0.0003 (10)
O1w	0.085 (3)	0.085 (3)	0.020 (3)	0.0425 (15)	0.000	0.000
O5	0.0129 (12)	0.0141 (12)	0.0106 (12)	0.0085 (11)	−0.0015 (10)	−0.0039 (10)

### Geometric parameters (Å, º)

**Table:**

Nd1—O2^i^	2.395 (2)	C4—C5	1.389 (5)
Nd1—O3^ii^	2.426 (2)	C4—C7	1.515 (5)
Nd1—O1^iii^	2.452 (2)	C5—N1	1.331 (4)
Nd1—O4	2.480 (2)	C5—H5	0.9300
Nd1—O5	2.482 (2)	C6—O3	1.256 (4)
Nd1—O5^iv^	2.485 (2)	C6—O4	1.264 (4)
Nd1—O5^v^	2.501 (2)	C7—O2	1.244 (4)
Nd1—N1	2.747 (3)	C7—O1	1.247 (4)
C1—N1	1.337 (4)	O1—Nd1^iii^	2.452 (2)
C1—C2	1.375 (5)	O2—Nd1^vi^	2.395 (2)
C1—C6	1.497 (4)	O3—Nd1^vii^	2.426 (2)
C2—C3	1.377 (5)	O5—Nd1^v^	2.485 (2)
C2—H2	0.9300	O5—Nd1^iv^	2.501 (2)
C3—C4	1.384 (5)	O5—H1	0.73 (4)
C3—H3	0.9300		
			
O2^i^—Nd1—O3^ii^	133.07 (8)	O5—Nd1—Nd1^v^	36.03 (5)
O2^i^—Nd1—O1^iii^	80.41 (9)	O5^iv^—Nd1—Nd1^v^	91.91 (6)
O3^ii^—Nd1—O1^iii^	77.55 (8)	O5^v^—Nd1—Nd1^v^	36.09 (5)
O2^i^—Nd1—O4	85.50 (9)	N1—Nd1—Nd1^v^	139.35 (5)
O3^ii^—Nd1—O4	78.54 (8)	Nd1^iv^—Nd1—Nd1^v^	68.623 (13)
O1^iii^—Nd1—O4	130.65 (8)	N1—C1—C2	122.8 (3)
O2^i^—Nd1—O5	81.79 (8)	N1—C1—C6	115.2 (3)
O3^ii^—Nd1—O5	131.58 (7)	C2—C1—C6	122.0 (3)
O1^iii^—Nd1—O5	77.64 (8)	C1—C2—C3	119.0 (3)
O4—Nd1—O5	146.31 (7)	C1—C2—H2	120.5
O2^i^—Nd1—O5^iv^	77.35 (8)	C3—C2—H2	120.5
O3^ii^—Nd1—O5^iv^	138.97 (8)	C2—C3—C4	119.2 (3)
O1^iii^—Nd1—O5^iv^	142.14 (8)	C2—C3—H3	120.4
O4—Nd1—O5^iv^	77.63 (8)	C4—C3—H3	120.4
O5—Nd1—O5^iv^	69.19 (8)	C3—C4—C5	117.8 (3)
O2^i^—Nd1—O5^v^	150.20 (8)	C3—C4—C7	121.6 (3)
O3^ii^—Nd1—O5^v^	69.18 (8)	C5—C4—C7	120.7 (3)
O1^iii^—Nd1—O5^v^	87.76 (8)	N1—C5—C4	123.4 (3)
O4—Nd1—O5^v^	122.15 (8)	N1—C5—H5	118.3
O5—Nd1—O5^v^	68.94 (8)	C4—C5—H5	118.3
O5^iv^—Nd1—O5^v^	96.63 (11)	O3—C6—O4	125.4 (3)
O2^i^—Nd1—N1	65.27 (8)	O3—C6—C1	116.8 (3)
O3^ii^—Nd1—N1	68.36 (8)	O4—C6—C1	117.8 (3)
O1^iii^—Nd1—N1	70.02 (8)	O2—C7—O1	125.7 (3)
O4—Nd1—N1	61.14 (8)	O2—C7—C4	116.5 (3)
O5—Nd1—N1	136.65 (8)	O1—C7—C4	117.8 (3)
O5^iv^—Nd1—N1	124.92 (8)	C5—N1—C1	117.8 (3)
O5^v^—Nd1—N1	135.26 (8)	C5—N1—Nd1	125.8 (2)
O2^i^—Nd1—Nd1^iv^	65.95 (6)	C1—N1—Nd1	116.4 (2)
O3^ii^—Nd1—Nd1^iv^	160.68 (5)	C7—O1—Nd1^iii^	133.4 (2)
O1^iii^—Nd1—Nd1^iv^	106.55 (6)	C7—O2—Nd1^vi^	142.0 (2)
O4—Nd1—Nd1^iv^	110.13 (5)	C6—O3—Nd1^vii^	148.7 (2)
O5—Nd1—Nd1^iv^	36.42 (5)	C6—O4—Nd1	126.1 (2)
O5^iv^—Nd1—Nd1^iv^	35.97 (5)	Nd1—O5—Nd1^v^	108.00 (8)
O5^v^—Nd1—Nd1^iv^	91.90 (6)	Nd1—O5—Nd1^iv^	107.49 (8)
N1—Nd1—Nd1^iv^	130.94 (6)	Nd1^v^—O5—Nd1^iv^	130.61 (10)
O2^i^—Nd1—Nd1^v^	114.28 (7)	Nd1—O5—H1	115 (3)
O3^ii^—Nd1—Nd1^v^	96.13 (6)	Nd1^v^—O5—H1	104 (3)
O1^iii^—Nd1—Nd1^v^	69.98 (5)	Nd1^iv^—O5—H1	91 (3)
O4—Nd1—Nd1^v^	155.32 (5)		

Symmetry codes: (i) −*x*+*y*−1/3, −*x*+1/3, *z*+1/3; (ii) *y*, −*x*+*y*, −*z*; (iii) −*x*+1/3, −*y*+2/3, −*z*+2/3; (iv) *x*−*y*, *x*, −*z*+1; (v) *y*, −*x*+*y*, −*z*+1; (vi) −*y*+1/3, *x*−*y*+2/3, *z*−1/3; (vii) *x*−*y*, *x*, −*z*.
